# Harnessing the Role of Three Lactic Acid Bacteria (LAB) Strains for Type II Sourdough Production and Influence of Sourdoughs on Bread Quality and Maillard Reaction Products

**DOI:** 10.3390/foods13121801

**Published:** 2024-06-07

**Authors:** Mustafa Sahin, Muhammed Ozgolet, Hasan Cankurt, Enes Dertli

**Affiliations:** 1 Department of Food Engineering, Faculty of Chemical and Metallurgical Engineering, Yildiz Technical University, Davutpasa Campus, Istanbul 34210, Turkey; mustafa_sahin_88@hotmail.com (M.S.); edertli@yildiz.edu.tr (E.D.); 2 Food Technology Department, Safiye Cikrikcioglu Vocational School, Kayseri University, Kayseri 38000, Turkey; hcankurt@kayseri.edu.tr

**Keywords:** sourdough bread, lactic acid bacteria, aroma profile, FAST index, Maillard reaction

## Abstract

This study tested the effect of *Companilactobacillus paralimentarius* E-106, *Lactiplantibacillus paraplantarum* N-15 and *Lactiplantibacillus plantarum* SC-9 on the amount of Maillard reaction and aroma profile in bread making with main bread quality parameters. The specific volumes of sourdough and control breads were in the range of 2.97–3.04 cm^3^/g, and the control II bread had the highest hardness values on all days. The FAST index value was determined to be between 40.48% and 81.22% in all breads. The FAST index value was found to be higher in the control breads than in the sourdough breads. In the volatile compounds analysis, 72 volatile compounds were detected. The variety of volatile compounds in the breads with sourdough addition was higher than the control breads. Among the tested strains, *Companilactobacillus paralimentarius* E-106 demonstrated superior properties for bread characteristics in comparison to other strains as a type II sourdough starter. In summary, improved aroma profile and decreased Maillard reaction products can be provided by sourdough addition without changing the bread quality, along with meeting consumer demand for less additive use.

## 1. Introduction

Cereals are recognized as easily accessible and inexpensive staple foods in terms of calories, protein and carbohydrates in numerous developing countries that do not have access to animal protein sources [1]. Among the cereal group products, the most consumed cereal product today is bread. The quality of bread is characterized by its taste, nutritional value, texture and shelf life. In the bakery industry, these properties are improved by the addition of bread additives or enzymes. Alternatively, the addition of sourdough affects all aspects of bread quality, thus meeting consumer demands for less additive use. Biochemical differences occur in the protein and carbohydrate components of the flour during the fermentation of sourdough. The rate and extent of these differences greatly affect the qualities of the sourdough and thus the quality of the bread dough and the bread structure. In addition, during sourdough formation bioactive compounds can be synthesized which might reduce the gastrointestinal syndromes and gluten sensitivity via decreasing the amount of causative grain-based ingredients [2]. It has been observed that when the pH of the dough decreased, suitable conditions were provided for the grain-based phytase to function and the mineral bioavailability increased [3]. Consumer demand for more nutritious, healthy, clean and artisanal-labeled products has increased the use of sourdough in bread making. Higher concentrations of organic acids are produced during sourdough production, particularly lactic acid and acetic acid, that might show antimicrobial activity, which can increase the shelf life of sourdough bread. Therefore, the use of sourdough in bread making allows the production of clean labeled products without using chemical preservatives. The effects of sourdough fermentation on the texture, taste, shelf life and nutritional value of food products were examined in many studies and its positive role in bread production was emphasized [4,5]. It has been observed that the type of leavening agents used (sourdough, commercial yeast, etc.) also affects Maillard reactions and the resulting products [6].

The sourdough processing process provides technical advantages in extending the texture (specific volume, pore structure, soft and flexible structure, color, crust thickness, crispness, crumbling, color), taste (flavor, inner and crust aroma) and shelf life [7]. The product quality of sourdough breads, which have remarkable health benefits compared to white bread, varies according to the sourdough used and the microbiota of this dough. However, the positive aspects of sourdough addition are not valid for all LAB strains and, importantly, strain-specific properties can affect the usability of LABs in sourdough systems. Many studies have demonstrated the addition of sourdough affected the technological properties, such as specific volume, and textural characteristics unfavorably [8,9]. Also, a few studies have focused on the effect on the advance of Maillard reactions [6,10]. Lower pH due to LAB activity increases the amount of free amino acids and reducing sugars in the medium due to the activated cereal amylases and proteases and the proteolytic activities of lactic acid bacteria. However, pH falling below the pKa values of amino groups causes the nucleophilic properties to decline, causing the initial step of the Maillard reaction to decrease. The decreasing pH increases the reactivity of reducing carbohydrates and also causes sugar degradation [11]. Therefore, the influence of the usage of distinct LAB strains for the formation of sourdough and the role of these sourdoughs on bread quality and the advance of Maillard reactions should be investigated. From this perspective, this study aimed to produce type II sourdoughs using sourdough isolates *Companilactobacillus paralimentarius* E-106, *Lactiplantibacillus paraplantarum* N-15 and *Lactiplantibacillus plantarum* SC-9, and mixtures thereof, to determine their roles in various quality characteristics of sourdough. For this, type II sourdoughs were characterized in terms of fermentation properties, and sourdough breads were tested in terms of specific volume, texture, color properties, nutritional composition, amount of Maillard reaction products (MRP), fluorescence of advanced MRPs, soluble tryptophan (FAST) index and volatile components. This study reveals the importance of strain selection for type II sourdough systems and shows the importance of sourdough technology for the reduction of MRPs.

## 2. Materials and Methods

### 2.1. Materials

The flour used in our bread-making process was obtained from a retail chain in Istanbul. The composition of flour was lipids (1.6%), carbohydrate (70.0%), sugar (0.5%), fiber (3.3%), protein (10.8%), salt (0.1%) and ash (max. 0.65%) at given ratios. The salt and pressed yeast (Pakmaya) used were also supplied by a retail chain in Istanbul. Drinkable tap water was used as the water. The LAB strains used as starter cultures were obtained from the culture collection of Yıldız Technical University, Department of Food Engineering. The strains used were *Companilactobacillus paralimentarius* E-106, *Lactiplantibacillus paraplantarum* N-15 and *Lactiplantibacillus plantarum* SC-9, which were all sourdough isolates.

### 2.2. Methods

#### 2.2.1. The Preparation of Sourdough with LAB Strains

The LAB strains were propagated from their glycerol stocks and further grown in 500 mL of MRS broth for 24 h at 30 °C. The resulting LAB cell suspension was centrifuged (2410 g, 10 min, 4 °C) to precipitate the cell pellet. After discarding the supernatant, the cells were diluted with distilled water and added to a water–flour mixture with dough yield of 170 to obtain 7–8 log cfu/g as the initial bacterial count (Supplementary Table S1). For the mix starter culture, the same microbial count was obtained as for a mixture of the 3 LAB strains. The doughs were incubated for 24 h in a climate chamber at 30 °C with 80% humidity [12].

#### 2.2.2. LAB Counts, Titratable Acidity and pH of Sourdough

LAB numbers in the sourdough samples were determined at the beginning (0 h) and at the end of the fermentation period (24 h). For this, ten grams of sourdough was dispersed in 90 mL of 0.9% NaCl solution and homogenized, and 0.1 mL of cell suspension was inoculated on MRS agar and streaked. The plates were then incubated at 30 °C for 48 h under anaerobic conditions and bacterial counts were given as log cfu/g [13].

The pH and titratable acidity (TTA) of the sourdough samples were measured at 0 and 24 h to monitor the pH drop and acid formation. The pH and acidity of the doughs were measured when they were first kneaded and just before they were baked. For this, ten grams of sourdough was dispersed in 90 mL of distilled water followed by mixing thoroughly, and the pH measurement was conducted with a pH meter (HANNA, Vöhringen, Germany). TTA was expressed as the amount of 0.1 N NaOH consumed to determine the amount of acid in a 10 g dough sample in mL [14].

#### 2.2.3. Preparation of Sourdough Breads

Sourdough breads were prepared with the sourdough samples produced with three distinct LAB strains with the formulation given in Supplementary Table S2. Control breads were prepared spontaneously without starter addition. Recipes were generated taking into account previous bread production trials. The ingredients in the formulation were added to the dough mixer. The dough was kneaded for 3 min at slow speed and then at high speed for 8 min. The dough was given a round shape at room temperature and rested for 15 min. It was then portioned into 177.5 g pieces. The dough, which was formed into a round shape, was rested for 5 min and then placed in cooking pots and fermented for 190 min at 30 °C in an 85% humidity environment in a proofing cabinet (Nuve TK252, Ankara, Turkey). The control II dough, which contained a higher amount of yeast than the other doughs, was fermented for 140 min in the proofing cabinet in order to simulate commercial production better, as mentioned above [15]. The oven top temperature was set at 205 °C and the bottom temperature at 195 °C. The breads were baked for 24 min in the electrical oven (Fimak, Istanbul, Turkey).

#### 2.2.4. Physical Characteristics of Bread Samples and Bake Loss

The rapeseed displacement method was used to determine the specific volume (SV) of the bread samples with a bread volume meter (Şimşek Laborteknik, Ankara, Turkey).

Digital images of the crumb were analyzed using ImageJ2x version 1.54c software (NIH, Bethesda, MD, USA, https://imagej.nih.gov/ij/) (accessed on 20 May 2024). The analysis entailed interpreting the images by assessing the contrast discrepancies between the pores and the solid phases. The images were first cropped, then converted to grayscale, and finally binarized once the threshold was reached to quantify the pore area and total pore area within the crumb (in square millimeters). The porosity was determined by estimating the percentage of pores within the total measured area [16]. Bake loss was calculated by measuring the weight of the bread dough before baking and then measuring the weight of the bread two hours after baking.

L*, a*, b* values of the crust and crumb color of the bread samples were measured by colorimeter (CR-100 Konica Minolta, Tokyo, Japan). L* is an index that gives information about brightness and darkness, where a* is positive for red tones and negative for green tones, and b* is positive for yellow tones and negative for blue tones [17]. Comparisons were made using the whiteness index (*WI*) of the inside of the bread and the brownness index (*BI*) of the outside of the bread [18]. The whiteness index was calculated using Equation (1) and the browning index using Equation (2).

(1) $$WI=\sqrt{{\left(100-L^{*}\right)}^{2}+a^{*2}+b^{*2}}$$

(2) $$BI=100-L^{*}$$

In these equations, L* is the brightness–darkness scale (black–white), a* is the red–green scale, b* is the yellow–blue scale. 

#### 2.2.5. Texture Profile of Bread Samples

In order to reveal the parameters of the texture profile analysis (TPA), each bread sample was pressed by TA.XT2 Plus texture analyzer (Stable Micro Systems Ltd., Godalming, UK) fitted with a 5 mm load cell and a 36 mm diameter cylindrical pressure probe, and the results were recorded. Slices 1.25 cm thick were cut from the bread and two slices were placed on top of each other in the test device. The analysis was conducted with the following settings: pretest speed 5.0 mm/s, test speed 1 mm/s, 30% compression, trigger type auto force 5 g, resting time between first and second compression 5 s. TPA was conducted on the day of baking (1st day), the 3rd day and the 5th day. The results are given as the average of three repetitions [19].

#### 2.2.6. Compositional Analysis of Bread Samples

The amount of protein in the bread samples was determined using the Kjeldahl method-based AACC Method No: 46-11.02 [20]. The nitrogen conversion factor used was 5.7 for the bread samples. Fat was determined using the Soxhlet method according to AACC Method 30-25.01 [20]. Ash content was found according to AACC Method No. 08-01.01 [20]. The bread samples were dried in a drying oven at 105 °C for 24 h for moisture determination. The carbohydrate value was found by subtracting the protein, fat, ash and moisture values from 100%.

#### 2.2.7. Maillard Reaction Products (FAST Index)

In studies of Maillard reactions (MR), fluorescence measurement is utilized for further detection of MRPs, which emit fluorescent light. The FAST index is frequently used to obtain fast and reliable results showing the advance of MRs. Therefore, the FAST index was used to evaluate MRPs in the bread samples. Extraction of fluorescent substances was performed following the procedure published in [21], with minor modifications. For this, 700 mg of homogenized bread sample was dissolved in 25 mL of 0.1 M borate buffer with a pH of 8.2. The soluble components were filtered through cellulose filter paper and used for fluorescence measurement. A fluorescence spectrophotometer (Photon Technology International, Lawrenceville, NJ, USA) was used for excitation and emission at 290 and 340 nm for tryptophan fluorescence (F_TRP_) and at 320 and 395 nm for advanced Maillard products fluorescence (F_AMP_). The FAST index was calculated as follows:

FAST index = (F_AMP_/F_TRP_) × 100 (3)

#### 2.2.8. Volatile Organic Compounds in Bread Samples

After the doughs were baked, the breads were taken out of the oven and kept in a dry indoor environment for 4 h. Volatile components of two control breads and four sourdough breads were detected by gas chromatography and mass spectroscopy (GC-MS) equipped with a Restec (Bellfonte, Bellefonte, PA, USA) Rtx-5MS fused silica capillary column (30 m × 0.25 mm × 0.25 µm). Solid phase micro-extraction (SPME) method was used in the preliminary preparation stage of the samples taken for testing by adapting the method described [22]. Bread samples of 2 g were weighed into a 20 mL headspace vial and incubated for 1 h at 60 °C. The GC column temperature program was set as indicated: 5 min waiting at 35 °C, then the temperature was increased to 50 °C with an increase of 5 °C/min and kept constant at 50 °C for 5 min. It was then set to 230 °C with an increase of 5.5 °C/min and kept at this temperature for 5 min and the analysis was concluded. The total run time for one sample was 50.73 min. Helium gas was preferred as the carrier gas and the flow rate was 1 mL/min. Mass spectra were scanned in the range of 35 to 650 masses at an ionization energy of 70 eV. In order to detect the volatile components, the spectra in the NIST27 and WILEY27 mass spectrum libraries and the spectra obtained from the analysis were compared; as a consequence, the volatile compounds were defined considering 70% or more similarity acceptable.

#### 2.2.9. Statistical Analysis

One-way analysis of variance was used to evaluate significant differences between sourdough samples and bread samples (ANOVA; *p* < 0.05, Tukey’s test). Statistical calculations were made using the IBM SPSS Statistics 26 program.

## 3. Results and Discussion

### 3.1. LAB Count, Titratable Acidity, and pH of Sourdough and Bread Doughs

The LAB numbers were determined in the sourdough samples before and after fermentation period, and the initial LAB numbers in the sourdough samples were around 7.17 log cfu/g. Following the 24 h fermentation period, the LAB counts were observed to be between 7.98 and 8.99 log cfu/g in the sourdough (Supplementary Table S3). In a previous study, the results of the LAB count of 12 sourdough samples collected from different bakeries were in the range of 8.35–8.96 log cfu/g [23]. In another study, 8.40 log cfu/g LAB was detected in a sourdough sample incubated at 30 °C for 15 h [24]. Similar LAB amounts in sourdoughs were found in other studies compared to the sourdough samples in this study.

For the TTA analysis, following the incubation process, the TTA values of sourdoughs prepared using three LAB strains were observed to be between 10.15 mL to 10.72 mL, whereas the control samples demonstrated 2.12 mL and 2.28 mL TTA values (Table 1). Following fermentation, the highest acidity was detected in the sourdoughs inoculated with *Companilactobacillus paralimentarius* E-106, while the lowest acidity was detected in the sourdough inoculated with *Lactiplantibacillus plantarum* SC-9 among the starter-containing sourdough samples. The decrease in pH value is caused by acids such as lactic acid and acetic acid, which occur as a result of LAB activity in sourdough. While the pH values of four different sourdoughs prepared with three different LAB strains were between 4.98 and 5.49 before fermentation, these values decreased to pH of 3.21–3.68 after fermentation (Table 1). In a study, the pH of sourdough prepared by *Lb. plantarum* inoculation decreased to 3.8 from 6.1 after incubation process. For the same sourdough, the TTA value increased from 1.5 mL to 12.8 mL [25]. In another study, the pH values of 12 different sourdoughs were measured and found to be between 3.37 and 3.95 [23]. These findings demonstrate that all three LAB strains were effective in terms of the desired acidity development in sourdough samples.

The TTA values of doughs and breads with added sourdough were found to be higher than the TTA values of both control breads and doughs (Table 2). Moreover, in the breads with LAB addition, lower pH values were observed before and after fermentation and after baking compared to the breads without LAB (Table 2). Among LAB-added samples, the lowest TTA before and after baking was found in the sample with *Companilactobacillus paralimentarius* E-106, while the highest TTA value was observed in the MIX-coded sample. The lowest pH value was observed in bread prepared with sourdough inoculated with *Lactiplantibacillus paraplantarum* N-15 (pH 4.60), while the highest pH value was found in the MIX-coded bread containing sourdough inoculated with three different strains (pH 4.89). The pH value of the control I bread, which had less commercial yeast and was fermented for a longer period, was found to be 6.05, whereas the pH value of the control II bread, which had a high amount of commercial yeast and was fermented for a shorter time, was 5.97. In another study, the TTA values were 6.8 mL and 8.6 mL while the final pH was 4.7 and 4.5 in breads prepared with the addition of 20% and 30% sourdough, respectively [26]. Similar pH levels were observed in bread samples prepared with sourdoughs fermented by *Lb. plantarum* and *Lb. brevis* [27]. In the present study, 12.7% of the total bread dough consisted of sourdoughs and the final pH was 4.60–4.89, while the TTA values were in the range of 3.42–4.29 mL in LAB-added sourdough breads. Although similar trends were observed in different studies in terms of sourdough and bread dough pH and TTA values, the final acidity of sourdough and bread dough prepared with sourdough addition depends on several factors such as strain type, fermentation period, sourdough amount and dough yield [7].

### 3.2. Physical Characteristics of Sourdough Breads

The specific volumes of sourdough breads produced in the present study were found to be between 2.97 and 3.04 cm^3^/g (Table 3). In terms of different groups, the specific volumes of the MIX starter and *Companilactobacillus paralimentarius* E-106-added breads were the highest, whereas the *Lactiplantibacillus paraplantarum* N-15-added and control I breads had the lowest specific volume. Previously it was reported that sourdough addition containing *Lb. plantarum* resulted in an increment of bread specific volume from 2.62 to 2.79 [25]. In the sourdough breads prepared by adding *Lb. paracasei* K5, the specific volume of the bread with 30% sourdough added was 2.5 cm^3^/g, while the volume of the bread with 20% sourdough added was 2.4 cm^3^/g [26]. As shown for strains SC-9 and E-106, as well as in other studies, the addition of sourdough resulted in an increase in bread volume. On the contrary, the specific volume of the sourdough bread N-15 demonstrated a lower specific volume. Previously, a high proteolytic activity as well as lower exopolysaccharide (EPS) production capabilities of sourdough isolate *Lb. paraplantarum* strains were reported [23], and it can be suggested that the lower specific volume of *Lactiplantibacillus paraplantarum* N-15-added breads might be attributed to the high level of proteolytic activity, potentially leading to gluten hydrolysis, as well as the low EPS production capability of strain N15.

**Figure 1:**
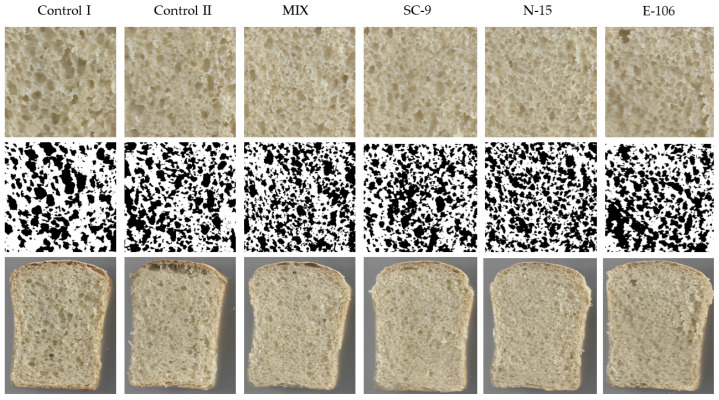
Crumb appearance and binarized images of bread samples using ImageJ2x version 1.54c.

The digital crumb appearance and binarized image of the muffins are presented in Figure 1. Through digital image analysis, the porosity and circularity of the crumb pores were computed. The porosity of the control I bread was lower compared to the other breads. It is probably due to a lack of LAB and yeast that produce CO_2_, which is the main cause of porosity. The bread with *Companilactobacillus paralimentarius* E-106 showed higher porosity among all breads, consistent with its higher specific volume. A perfect circle is indicated by a circularity rating of 1.0. An increasingly elongated shape is indicated as the value gets closer to 0.0 [28]. The circularity of crumb pores ranged from 0.728 to 0.766, and the circularity of the MIX-coded bread crumb was higher than the SC-9-coded bread crumb, whereas the other bread crumb pores were not different from each other significantly (*p* > 0.05). Santos et al. [29] found the circularity of white bread with Baker’s yeast and sourdough bread as 0.77 and 0.81, respectively. The control I bread had a higher bake loss compared to control II and the MIX-coded bread. It may be attributed to increased protein/starch complexes due to pH decrease [30]. There is no significant difference among the other samples statistically (*p* > 0.05).

Color evaluation of the breads was carried out considering their L*, a*, b* values. The WI for bread crumbs and BI for bread crust were calculated and are presented in Table 3. The breads with the highest WI were the control I and control II breads. There was no significant difference between the breads with added sourdough, except for the sourdough bread with *Lactiplantibacillus plantarum* SC-9. Similarly, the WI of sourdough bread was lower compared to white bread without sourdough addition [31]. The highest value in BI was determined in the control I and control II breads. In our study, the advance of MRs was demonstrated via the FAST index, which is an indicator of MRPs. The FAST indexes of the control breads were found to be higher than the breads with added sourdough. The BI of sourdoughs were differentiated from each other, which is attributed to the advance of MRs and caramelization in the course of baking depending upon the metabolic activity of LAB during fermentation. Yezbick et al. [32] also found that the L* value of sourdough-added soy bread was higher compared to bread without sourdough, which displays lower BI. Caglar et al. [33] stated that the browning indexes of seven out of eight sourdough breads (32.3–47.4) were found to be lower than control breads (45.4). As a consequence, the whiteness and browning indexes of the breads in our study were consistent with the values in the literature.

### 3.3. Textural Properties of Sourdough Breads

**Figure 2:**
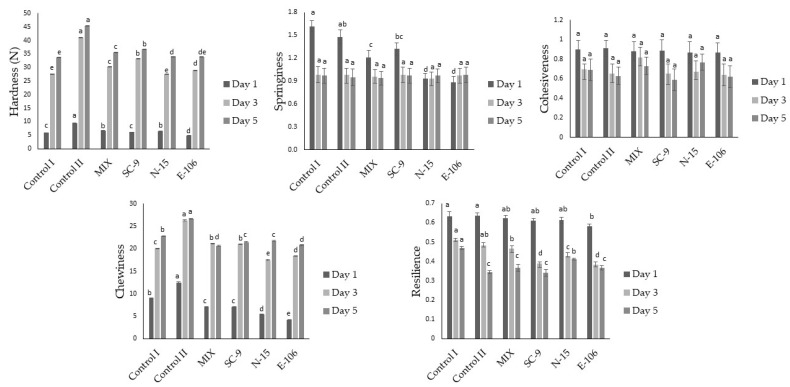
Textural properties of breads on day 1, 3 and 5. Different small letters on the columns show the significant difference between the breads for each textural parameter prepared with or without LAB starter (*p* < 0.05).

The textural attributes evaluated in this study were hardness, springiness, cohesiveness, chewiness and resilience, and the results are presented in Figure 2. Hardness describes the force required to compress food between the palate and tongue or between the teeth. Springiness is the ability to recover after initial compression. Adhesiveness describes the internal forces within the food that prevent sample fragmentation. Chewiness measures the energy required to make food suitable for swallowing. Resilience is the resistance of food to returning to its original shape and size after pressure has been applied [34]. While the bread with the lowest hardness value on day 1, 3 and 5 was *Companilactobacillus paralimentarius* E-106-added bread, the control II bread was the bread with the highest hardness on day 1, 3, and 5. E-106-coded sourdough bread had the highest specific volume, which is consistent with the hardness since specific volume is negatively correlated with hardness value typically. The highest hardness value among the sourdough breads with a starter was found for the MIX and *Lactiplantibacillus paraplantarum* N-15-added sourdough breads on day 1; however, the *Lactiplantibacillus plantarum* SC-added bread had the highest hardness on day 5. In a previous study, the hardness value in bread obtained from sourdough was determined as 5.7 N, which is close to the values in our study [25]. In another study, the texture of different breads was examined and the lowest hardness was provided in breads with 22.5% sourdough addition, while the highest hardness was determined in bread without sourdough addition [35]. Since biological acidification, EPS and dextran formation give rise to improved moisture distribution and higher specific volume among sourdough breads, softer bread characteristics can be formed [36]. The springiness of the control I and control II breads on day 1 was higher than for the sourdough breads. Casado et al. [37] also found that the springiness of sourdough bread was higher compared to non-sourdough bread. However, no significant differences were observed between all breads in terms of springiness values on the 3rd and 5th days. In addition, the breads had no significant difference in terms of adhesiveness on day 1, 3 and 5, even though the adhesiveness showed decreasing trends with storage. In contrast, the chewiness of the breads increased during storage. The chewiness values of the control I and II breads on the 1st day were higher than the sourdough breads; however, on day 3 and 5, the control II bread had the highest chewiness values. Jitrakbumrung et al. [35] also stated that breads without sourdough addition offered higher chewiness values. The breads with relatively higher resilience values on the 1st day were control I and II. On the 3rd and 5th day, the highest resilience was observed in the control I bread. The resilience values of the breads also decreased during storage. Consistent with our study, Crowley et al. [38] stated that hardness and chewiness increased and resilience decreased with storage time for both control and sourdough breads. In the same study, hardness and chewiness decreased with 20% sourdough addition, but 40% sourdough addition led to firmer bread.

### 3.4. Chemical Composition of Sourdough Breads

The breads were analyzed to determine their chemical composition (Table 4). The protein, fat and ash values of the bread samples were found to be between 6.77 and 6.94%, 0.246 and 0.269%, and 0.5815 and 0.6084%, respectively, and no significant difference was observed for these components among the bread samples (*p* < 0.05). In terms of moisture value, it was determined that the moisture value of control II was the lowest followed by control I. Usage of sourdough starters and sourdough bread preparation resulted in an increase in the moisture content of the bread samples. Previously, the role of sourdough technology in the enhanced moisture content in sourdough breads was attributed to the acidification and reduction of glutenin disulfide bonds by heterofermentative LAB which could promote the work of proteases with decreasing pH. As a result of increased proteolytic activity with the effect of proteases, more hydrophilic short-chain peptides and free amino acids could be formed, which might lead to increased water retention in the bread [39]. The results of the moisture analysis on the 1st day were also supported by the hardness values determined on the 1st day, as the increase in moisture brings about softer bread. In our study, the carbohydrate values in the breads varied between 44.55% and 45.15%. While the amount of carbohydrate in the control II bread was 45.15%, it was determined as 44.55% in the bread containing *Lactiplantibacillus plantarum* SC-9 isolate. It has been observed that the amount of starch in breads with sourdough addition can be different. This difference is thought to be due to strain-specific properties. Sugars consumed by LAB can cause a decrease in the total amount of carbohydrates. Starch hydrolysis due to increased alpha-amylase activity and the resulting conversion of hydrolysis products to other products such as lactic acid, acetic acid and alcohol can be shown as the reason for this decrease [4,40].

### 3.5. Maillard Reaction Products (FAST Index) of Sourdough Breads

Fluorescence measurements at different wavelengths, and the FAST index, were used to evaluate the progress of MRs. In many model studies and food products, fluorescent MRPs have been interpreted to evaluate the rate and amount of MRs [10]. The FAST index is a fast, precise, low-cost methodology to measure the degree of MRs by the fluorescence of advanced Maillard products (AMP) such as pyrrole and imidazole derivatives. In our study, the FAST index of the control group breads was found to be higher than the breads with added sourdough (Table 5). The FAST index values of all breads were between 40.48% and 81.22%. While the highest FAST index value was found in the control II bread (81.22%) followed by the control I bread (71.17%), the lowest FAST index value was found in the MIX-coded sourdough bread (40.48%). Among the sourdough breads, the highest FAST index value was found in the bread containing sourdough with the addition of *Companilactobacillus paralimentarius* E-106 isolate (59.71%). The intensity of MR depends on various parameters such as pH, type and concentration of free amino acids, type and concentration of sugars, temperature, presence of buffer and water activity [41]. The decrease in pH causes the MR rate to decrease [42]. When sourdough was used with commercial yeast, a decrease was observed in the amount of CML, an advanced glycation product, as a result of the acidification of the dough [43]. This change that may occur in the amount of MR is based on the fact that the amino acids produced and consumed by LAB are different from those of yeast. The FAST index values of sourdough breads were observed to be different and this difference might be due to the amount of amino acids and sugars that LAB produce and consume as a result of their metabolic activities [10]. Excessive MRP consumption is an independent risk factor for chronic oxidative stress and inflammatory factor surges in adulthood. Therefore, sourdough addition has a potential to decrease the health risks associated with MRPs.

### 3.6. Volatile Organic Compounds of Sourdough Breads

**Figure 3:**
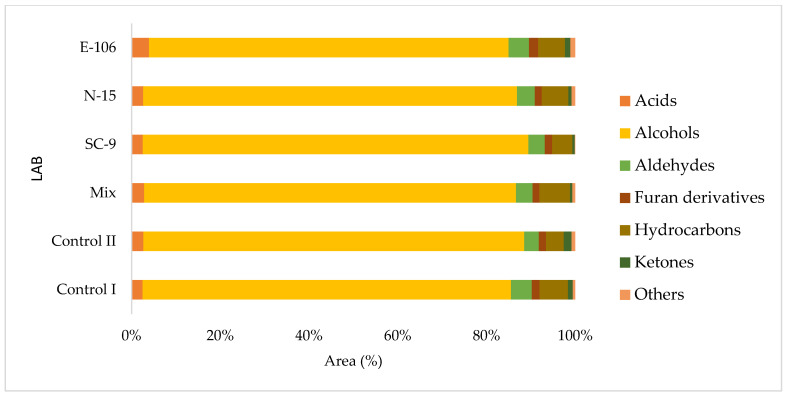
The levels of volatile organic compound groups for each bread relative to peak area (%).

Volatile compounds during fermentation mainly originate from microbial and yeast metabolism, enzymatic reaction or oxidation of their lipids, and MRs. Volatile component profiles of the bread samples were determined by solid phase microextraction (SPME) method and gas chromatography mass spectroscopy (GC-MS). As a result of the SPME/GC-MS analysis, 72 different volatile components were detected in the breads (Supplementary Table S4). The molecules with a similarity rate of less than 70% were not evaluated. The fermentation process is the main cause of volatile component formation in sourdough bread. It mainly causes the formation of acids, alcohols, aldehydes, esters and ketones. The proportions of organic compounds groups for each bread were illustrated in Figure 3. When our study was examined, aldehyde formation in breads with added sourdough was higher than in the control II bread. Troadec et al. [6] also found that the aldehyde amount is higher in sourdough bread compared to yeast-leavened bread, attributing this to the transformation of α-amino acids into structurally related aldehydes by Strecker degradation. The highest formation of aldehyde compounds among our sourdough-added breads was observed in the *Companilactobacillus paralimentarius* E-106-added sourdough bread. In addition, the bread containing *Companilactobacillus paralimentarius* E-106-added sourdough generated a higher amount of ketones than the other starter-added sourdough breads. When examined in terms of acid compounds, the total acid components were found to be higher in breads with sourdough addition than in the control group breads. Plessas et al. [22] compared the organic acid amounts of three types of sourdough breads and one control bread. According to this study, the total amount of acids was found to be higher in sourdough added breads than in the control bread. In our study, the amount of alcohol was found to be higher in breads prepared with sourdough. In a study, high alcohol concentrations were generally higher in breads with added sourdough than in breads without added sourdough. In this way, it has been stated that sourdough fermentation also contributes to the bread flavor profile [44]. Different combinations of LAB and yeast affect the production of volatile compounds and the aromatic flavor of breads. Mixed cultures of LAB and yeasts contribute to the production of complex volatile compounds compared to single-culture starters. Alcohols constitute the main group of volatile compounds in the control and sourdough breads. The total alcohol and ethanol content were higher in breads with added sourdough compared to the control I group. While the highest total alcohol amount was detected in bread containing *Lactiplantibacillus plantarum* SC-9 isolate among breads with added sourdough, the lowest total alcohol amount was detected in bread containing *Companilactobacillus paralimentarius* E-106. In another study, it was observed that the amount of alcohol formed in sourdoughs in which LAB and yeast strains were added was higher than in dough formed by inoculation of only yeast [45]. When the yeast is added to the dough, the yeast starts to utilize the maltose in the mixture, forming sugar, alcohol and carbon dioxide, but if the mixture is left to ferment for longer periods, the acid formed by the oxidation of the alcohol causes the product to turn sour, which may cause a decrease in the amount of alcohol. In another study, the level of volatile compounds derived from fermentation was linked to the level of yeast compared to the fermentation time [46]. Yeasts break down the sugars in foods and turn them into CO_2_ and ethanol. Therefore, the increased total alcohol content of the control II bread in comparison to the control I bread might be due to the higher yeast content of the control II bread. The total hydrocarbon content of the control group breads was lower than the breads with sourdough addition. The amount of hydrocarbons in sourdough-added bread produced with the MIX starter was found to be higher than all other breads. An increase in the amount of hydrocarbons was observed during whole wheat sourdough fermentation [47]. When our study is examined in terms of furan derivatives, sourdough addition resulted in an increase in comparison to the control group breads. Mietton et al. [48] also stated that sourdough addition led to higher furan derivatives and displayed a more diverse aroma profile.

## 4. Conclusions

The highest specific volume value was seen in bread using the *Companilactobacillus paralimentarius* E-106 strain. In general, the addition of sourdough did not cause a negative effect on the bread volume. The lowest hardness and chewiness values were observed in bread using the *Companilactobacillus paralimentarius* E-106 strain. The lowest chewiness among all the breads was determined in breads with sourdough addition. The moisture content of starter added sourdough breads was found to be higher than control bread, which may cause softer bread, which is supported by hardness values. It has been observed that the addition of sourdough improves the bread flavor profile. The formation of aldehydes, alcohols, acids hydrocarbons and furan derivatives were detected at higher levels in sourdough breads. The advance of MRs was lower in sourdough bread, which was indicated via the FAST index. The browning index was also lower in sourdough, probably due to lower formation of brown-colored MRs in sourdough breads. The reduction of MRPs may lead to a decrease in toxic compounds derived from MRs. In future studies, the amounts of hazardous compounds such as acrylamide, HMF and AGE in sourdough breads produced with these cultures will be examined. Thus, a healthier bread production was achieved via sourdough addition in this study without compromising the physicochemical and organoleptic properties of bread.

## Figures and Tables

**Table 1:** pH and TTA of sourdoughs before and after incubation.

	TTA		pH	
LAB	0 h	24 h	0 h	24 h
Control I	1.37 ± 0.08 ^b^	2.12 ± 0.03 ^d^	6.42 ± 0.02 ^b^	6.01 ± 0.05 ^b^
Control II	1.33 ± 0.03 ^b^	2.28 ± 0.03 ^d^	6.51 ± 0.02 ^a^	6.04 ± 0.05 ^a^
MIX	2.02 ± 0.03 ^a^	10.45 ± 0.09 ^b^	5.20 ± 0.02 ^e^	3.43 ± 0.01 ^e^
SC-9	2.13 ± 0.06 ^a^	10.15 ± 0.10 ^c^	4.98 ± 0.04 ^f^	3.21 ± 0.02 ^f^
N-15	2.05 ± 0.05 ^a^	10.35 ± 0.05 ^b^	5.49 ± 0.01 ^c^	3.68 ± 0.02 ^c^
E-106	2.03 ± 0.03 ^a^	10.72 ± 0.10 ^a^	5.38 ± 0.02 ^d^	3.55 ± 0.03 ^d^

Different small letters in the same column show the significant difference between the doughs prepared with or without LAB starter (*p* < 0.05). TTA: Total titratable acidity.

**Table 2:** Dough pH and TTA values before and after fermentation and after baking.

	pH			TTA		
LAB	First Dough	Last Dough	Bread Dough	First Dough	Last Dough	Bread Dough
Control I	6.53 ± 0.07 ^a^	6.14 ± 0.04 ^a^	6.05 ± 0.02 ^a^	1.10 ± 0.05 ^c^	1.92 ± 0.03 ^d^	1.82 ± 0.06 ^d^
Control II	6.42 ± 0.03 ^b^	6.09 ± 0.03 ^b^	5.97 ± 0.02 ^b^	1.13 ± 0.03 ^c^	1.93 ± 0.03 ^d^	1.83 ± 0.07 ^d^
MIX	5.54 ± 0.07 ^c^	4.97 ± 0.08 ^c^	4.89 ± 0.04 ^c^	2.33 ± 0.03 ^a^	4.48 ± 0.08 ^a^	4.29 ± 0.07 ^a^
SC-9	5.48 ± 0.04 ^e^	4.74 ± 0.06 ^e^	4.68 ± 0.03 ^e^	2.27 ± 0.03 ^a^	4.08 ± 0.06 ^b^	3.95 ± 0.03 ^b^
N-15	5.51 ± 0.03 ^d^	4.68 ± 0.04 ^f^	4.60 ± 0.04 ^f^	2.13 ± 0.03 ^b^	4.47 ± 0.08 ^a^	4.27 ± 0.04 ^a^
E-106	5.31 ± 0.01 ^f^	4.76 ± 0.04 ^d^	4.69 ± 0.03 ^d^	2.12 ± 0.03 ^b^	3.72 ± 0.03 ^c^	3.42 ± 0.06 ^c^

Different small letters in the same column show the significant difference between the doughs prepared with or without LAB starter (*p* < 0.05). LAB: Lactic acid bacteria. TTA: Total titratable acidity.

**Table 3:** Physical characteristics of bread samples and bake loss.

LAB	Specific Volume (cm^3^/g)	Bake Loss (%)	Porosity (%)	Circularity	WI (Crumb)	BI (Crust)
Control I	2.99 ± 0.04 ^cd^	13.07 ± 0.11 ^a^	40.69 ± 0.44 ^d^	0.753 ± 0.006 ^ab^	34.08 ± 0.10 ^a^	36.09 ± 0.19 ^a^
Control II	3.01 ± 0.05 ^bc^	12.39 ± 0.23 ^b^	44.74 ± 1.05 ^c^	0.752 ± 0.011 ^ab^	33.54 ± 0.60 ^a^	35.92 ± 0.19 ^a^
MIX	3.03 ± 0.07 ^ab^	12.15 ± 0.38 ^b^	45.58 ± 0.52 ^bc^	0.766 ± 0.012 ^a^	31.89 ± 0.08 ^b^	34.22 ± 0.09 ^c^
SC-9	3.01 ± 0.06 ^bc^	12.64 ± 0.03 ^ab^	46.38 ± 0.78 ^abc^	0.728 ± 0.009 ^b^	30.20 ± 0.06 ^c^	32.27 ± 0.12 ^d^
N-15	2.97 ± 0.06 ^d^	12.75 ± 0.09 ^ab^	47.42 ± 0.35 ^ab^	0.744 ± 0.012 ^ab^	32.08 ± 0.31 ^b^	35.12 ± 0.14 ^b^
E-106	3.04 ± 0.04 ^a^	12.66 ± 0.38 ^ab^	47.71 ± 1.04 ^a^	0.754 ± 0.012 ^ab^	32.05 ± 0.63 ^b^	32.80 ± 0.48 ^d^

Different small letters in the same column show the significant difference between the breads prepared with or without LAB starter (*p* < 0.05).

**Table 4:** Chemical composition and specific volumes of bread samples.

Bread Type	Protein (%)	Fat (%)	Ash (%)	Moisture (%)	Carbohydrate (%)
Control I	6.85 ± 0.10 ^a^	0.257 ± 0.003 ^a^	0.602 ± 0.008 ^a^	47.30 ± 0.10 ^cd^	45.01 ± 0.19 ^a^
Control II	6.83 ± 0.09 ^a^	0.246 ± 0.010 ^a^	0.606 ± 0.019 ^a^	47.19 ± 0.04 ^d^	45.15 ± 0.09 ^a^
MIX	6.85 ± 0.11 ^a^	0.253 ± 0.001 ^a^	0.605 ± 0.010 ^a^	47.36 ± 0.01 ^c^	44.95 ± 0.10 ^ab^
SC-9	6.94 ± 0.05 ^a^	0.259 ± 0.010 ^a^	0.582 ± 0.045 ^a^	47.7 ± 0.01 ^a^	44.55 ± 0.06 ^c^
N-15	6.81 ± 0.03 ^a^	0.254 ± 0.014 ^a^	0.608 ± 0.019 ^a^	47.68 ± 0.01 ^a^	44.66 ± 0.04 ^bc^
E-106	6.77 ± 0.09 ^a^	0.260 ± 0.013 ^a^	0.587 ± 0.031 ^a^	47.56 ± 0.02 ^b^	44.85 ± 0.09 ^ab^

Different small letters in the same column show the significant differences between the protein, fat, ash, moisture and carbohydrate amounts of different bread samples (*p* < 0.05).

**Table 5:** TRP fluorescence, AMP fluorescence and FAST index values.

LAB	TRP Fluorescence (290/340 nm)	AMP Fluorescence (340/420 nm)	FAST Index (%)
Control I	5866.44 ± 64.59 ^e^	4175.27 ± 58.44 ^c^	71.17 ± 0.80 ^b^
Control II	5986.11 ± 52.44 ^e^	4862.04 ± 76.14 ^b^	81.22 ± 0.79 ^a^
MIX	8438.21 ± 94.53 ^b^	3415.15 ± 69.01 ^d^	40.48 ± 0.99 ^f^
SC-9	11,201.59 ± 80.30 ^a^	5240.63 ± 72.46 ^a^	46.79 ± 0.98 ^e^
N-15	7597.42 ± 19.50 ^d^	4126.39 ± 62.15 ^c^	54.31 ± 0.95 ^d^
E-106	8092.97 ± 84.43 ^c^	4833.05 ± 95.84 ^b^	59.71 ± 0.61 ^c^

Different small letters in the same column show the significant difference between the breads prepared with or without LAB starter (*p* < 0.05). TRP: Tryptophan, FIC: Fluorescence intermediary compounds, FAST index: Fluorescence of advanced Maillard reaction products and soluble tryptophan. TRP fluorescence is a measure of fluorescence intensity (FI) at λextinction = 290 nm and λemmision = 340 nm. AMP fluorescence is a measure of fluorescence intensity (FI) at λextinction = 340 nm and λemmision = 420 nm. FAST index is the ratio of FI of TRP and AMP multiplied by 100.

## Data Availability

The original contributions presented in the study are included in the article/Supplementary Materials; further inquiries can be directed to the corresponding author.

## Supplementary Materials

The following supporting information can be downloaded at: https://www.mdpi.com/article/10.3390/foods13121801/s1, Supplementary Table S1: The formulation of prepared sourdoughs; Table S2: The formulation of prepared breads; Table S3: LAB counts in sourdoughs; Table S4: Volatile organic compounds profile of bread samples.
