# Supplementary material for: Harnessing the Role of Three Lactic Acid Bacteria (LAB) Strains for Type II Sourdough Production and Influence of Sourdoughs on Bread Quality and Maillard Reaction Products
Source: Foods. 2024 Jun 7;13(12):1801. doi: 10.3390/foods13121801 (PMC11202766; doi:10.3390/foods13121801)
Supplement: Supplementary file 1 [file foods-13-01801-s001.zip › foods-3012080-supplementary.pdf]

Table S1 The formulations of prepared sourdoughs

Ingredients	Added amount (g)	Sourdoughs			
	Control II	Mix	SC-9	N-15	E-106
**Flour**	100	100	100	100	100
**Water**	70	70	70	70	70
**SC-9 culture**	-	0.66	2	-	-
**N-15 culture**	-	0.66	-	2	-
**E-106 culture**	-	0.66	-	-	2
**Total water/ Total flour**	0.7	0.7	0.7	0.7	0.7

Table S2 The formulations of prepared breads

Ingredients	Added amount (g)					
	Control II	Control I	Mix	SC-9	N-15	E-106
**Flour**	100	100	100	100	100	100
**Water**	70	70	70	70	70	70
**Yeast**	1	0.56	0.56	0.56	0.56	0.56
**Salt**	1.5	1.5	1.5	1.5	1.5	1.5
**Sourdough**	25	25	25	25	25	25
**Total water/ Total flour**	0.7	0.7	0.7	0.7	0.7	0.7

Table S3 LAB counts in sourdoughs

	Sourdough (log cfu/g dough)	
**LAB**	**0.h**	**24.h**
**MIX**	7.92 ± 0.40^{c}	8.79 ± 0.06^{c}
**SC-9**	8.07 ± 0.44^{b}	8.97 ± 0.27^{b}
**N-15**	8.36 ± 0.24^{a}	8.99 ± 0.10^{a}
**E-106**	7.17 ± 0.33^{d}	7.98 ± 0.18^{d}

* Different small letters in the same column show the significant difference between LAB ($p < 0.05$).
LAB: Lactic acid bacteria

Table S4 Volatile organic compound profile of bread samples

Compounds	Retention time	Control I	Control II	Mix	SC-9	N-15	E-106
**Acids**							
**1,1'-bibicyclo (2.2.2)octyl-4-carboxylic acid**	1.96	2.03±0.03^{b}	2.22±0.04^{c}	2.22±0.03^{c}	2.13±0.02^{d}	2.34±0.02^{b}	2.89±0.01^{a}
**Iso-Valeric Acid**	6.49	-	-	0.10±0.03^{c}	0.18±0.02^{a}	-	0.12±0.02^{b}
**Propanedioic acid, dimethyl-**	13.85	-	-	0.23±0.05^{a}	-	-	0.20±0.03^{a}
**Acetic acid**	14.09	-	-	-	-	-	0.19±0.03
**Butanoic acid, 2-methyl-**	38.41	-	-	0.09±0.02^{a}	-	-	0.11±0.02^{a}
	**Total**	**2.03**	**2.22**	**2.64**	**2.30**	**2.34**	**3.51**
**Alcohols**							
**Ethanol**	2.19	61.82±0.72^{d}	63.7±0.82^{a}	67.81±0.94^{bc}	69.48±0.47^{b}	66.11±0.33^{c}	63.05±0.74^{d}
**1-Propanol, 2-methyl-**	3.19	0.34±0.05^{c}	0.51±0.0.1^{a}	0.29±0.03^{c}	0.30±0.03^{c}	0.23±0.01^{d}	0.40±0.01^{b}
**Isoamylalcohol**	4.61	3.67±0.05^{e}	4.10±0.02^{d}	5.99±0.06^{b}	5.72±0.03^{c}	6.19±0.05^{a}	6.19±0.03^{a}
**1-Hexanol**	6.99	1.56±0.02^{f}	1.61±0.04^{e}	2.07±0.04^{a}	1.93±0.03^{b}	1.75±0.03^{d}	1.83±0.03^{c}
**Heptanol**	8.86	-	-	0.19±0.03^{a}	0.13±0.02^{b}	-	0.22±0.04^{a}
**1-Octen-3-ol**	9.07	-	0.35±0.03^{c}	0.45±0.02^{b}	0.24±0.05^{d}	0.29±0.01^{d}	0.60±0.05^{a}
**2-Ethyl Hexanol**	10.23	-	0.09±0.00^{c}	0.14±0.01^{b}	0.17±0.01^{a}	-	0.13±0.02^{b}
**E,E-2,4-decadienol**	13.79	0.30±0.05	-	-	-	-	-
**Cyclohexanol, 1-methyl-4-(1-methylethenyl)-**	19.46	-	-	-	0.15±0.04	-	-
**(+)-Caran-Cis-4-Ol**	19.47	0.24±0.03	-	-	-	-	-
	**Total**	**67.93**	**71.31**	**76.94**	**78.12**	**74.57**	**72.42**
**Aldehydes**							
**Butanal, 3-methyl-**	2.10	-	-	0.12±0.01	-	-	-
**Hexanal**	5.70	1.03±0.05^{a}	0.69±0.02^{e}	0.78±0.03^{b}	0.68±0.02^{e}	0.99±0.05^{a}	1.06±0.06^{a}
**2-Octenal**	7.56	-	-	-	-	--	0.25±0.04
**Heptanal**	7.57	0.61±0.05^{a}	0.25±0.01^{b}	0.17±0.01^{d}	0.21±0.02^{c}	0.22±0.03^{c}	0.20±0.02^{c}
**Nonenal**	7.90	-	-	0.27±0.03	-	-	-
**2-Heptenal**	8.62	-	-	0.15±0.02^{a}	0.10±0.01^{b}	0.14±0.01^{a}	0.12±0.02^{a}
**Benzene acetaldehyde**	8.75	-	-	-	0.42±0.05^{a}	-	0.23±0.04^{b}
**Octanal**	9.59	0.21±0.03^{b}	0.24±0.04^{b}	0.09±0.01^{c}	0.19±0.04^{b}	0.48±0.04^{a}	0.44±0.05^{a}
**Benzaldehyde**	10.73	-	-	0.10±0.02^{a}	0.10±0.01^{a}	-	-
**Nonanal**	12.21	1.77±0.04^{a}	1.57±0.03^{b}	1.55±0.03^{b}	1.46±0.04^{c}	1.74±0.03^{a}	1.49±0.02^{c}
**2-Nonenal**	13.77	-	-	-	0.16±0.01	-	-
**Decanal**	15.01			0.20±0.03^{c}	-	0.28±0.03^{a}	0.32±0.03^{a}
**Undecanal**	15.03	0.22±0.02	-	-	-	-	-
	**Total**	**3.84**	**2.75**	**3.43**	**3.32**	**3.52**	**4.11**

**Furan derivatives**							
**2H-Pyran, 2-ethenyltetrahydro-2,6,6-trimethyl-**	5.71	-	-	-	-	-	0.33±0.01
**Furan, 2-pentyl-**	9.35	1.45±0.03^{b}	1.34±0.03^{c}	1.38±0.02^{c}	1.51±0.01^{a}	1.39±0.03^{c}	1.50±0.01^{a}
	**Total**	**1.45**	**1.34**	**1.38**	**1.51**	**1.39**	**1.83**
**Hydrocarbons**							
**Heneicosane**	3.07	-	0.51±0.04^{b}	-	-	-	0.68±0.03^{a}
**Dodecane**	4.12						0.57±0.04
**Cyclohexane, 1,1'-(1-methyl-1,3-propanediyl)bis**	5.01	0.32±0.04^{b}	-	-	-	-	0.38±0.03^{a}
**Pentane, 1-chloro-**	5.18	-	0.17±0.02^{b}	0.23±0.02^{a}	-	0.16±0.03^{b}	0.24±0.02^{a}
**Decane, 5-propyl-**	8.87	-	-	0.98±0.05	-	-	-
**Copaene**	9.13	-	-	0.08±0.01	-	-	-
**Naphthalene, 1,2,3,5,6,7,8,8a-octahydro-1,8a-dimethyl-7-(1-methylethenyl)-, [1R-(1.alpha.,7.beta.,8a.alpha.)]-**	9.48	-	-	0.17±0.02	-	-	-
**Gamma.-Terpinene**	9.86	-	0.08±0.01	-	-	-	-
**.Delta.3-Carene**	9.87	-	-	-	0.17±0.01	-	-
**Hexadecane**	10.21	0.84±0.04^{a}	-	0.69±0.04^{b}	-	-	-
**L-Limonene**	10.31	0.64±0.03^{b}	0.34±0.01^{d}	1.32±0.04^{a}		0.47±0.03^{c}	0.47±0.03^{c}
**Dodecane, 2,6,10-trimethyl-**	10.32	-	-	0.14±0.01	-	-	-
**D-Limonene**	10.34	-	-	-	1.28±0.05	-	-
**Dodecane, 2-methyl-**	10.37	-	-	-	-	-	0.23±0.02
**Nonane, 3-methyl-5-propyl-**	14.83	-	0.34±0.02^{c}	-	0.39±0.01^{b}	0.59±0.03^{a}	-
**Dodecane, 2,6,11-trimethyl-**	14.84	0.47±0.01^{b}	0.44±0.03^{b}	0.55±0.02^{a}	0.21±0.03^{c}	0.10±0.01^{d}	-
**Tetradecane**	20.69	1.87±0.02^{b}	1.23±0.03^{e}	1.42±0.04^{c}	1.30±0.02^{d}	1.50±0.05^{c}	2.49±0.06^{a}
**1,4-Methanoazulene, decahydro-4,8,8-trimethyl-9-methylene-,**	21.22	-	-	-	0.11±0.01^{b}	0.25±0.02^{a}	-
**Decane, 2,3,4-trimethyl-**	22.55	-	-	-	-	0.18±0.01	-
**Heptadecane, 2,6,10,15-tetramethyl-**	23.67	-	-	-	-	0.58±0.03	-
**Valencene**	23.79	-	-	-	0.12±0.02	-	-
**Pentadecane, 2,6,10,14-tetramethyl-**	25.02	-	-	0.27±0.03^{a}	-	0.20±0.02^{b}	-
**9-methylnonadecane**	25.18	-	-	-	-	0.21±0.02	-

**Octadecane, 5-methyl-**	25.18	0.50±0.03[a]	-	0.21±0.03[b]	-	-	-
**Pentadecane, 8-hexyl-**	25.68	-	0.22±0.02[a]	-	-	0.16±0.02[b]	-
**Hexadecane, 2,6,10,14-tetramethyl-**	26.47	0.27±0.03[c]	-	0.27±0.03[c]	0.50±0.04[b]	0.73±0.02[a]	0.24±0.03[c]
**Hexane, 1,6-dicyclohexyl-**	26.59	-	-	-	-	0.20±0.02	-
**Hexadecane, 1-iodo-**	29.07	0.34±0.01	-	-	-	-	-
**Tetracosane**	38.31	-	-	-	-	-	0.12±0.01
	**Total**	**5.25**	**3.33**	**6.33**	**4.08**	**5.33**	**5.42**
**Ketones**							
**2-Butanone, 3-hydroxy-**	4.23	0.23±0.02[d]	0.71±0.04[b]	0.29±0.02[c]	0.29±0.02[c]	0.29±0.01[c]	0.87±0.03[a]
**5-Hydroxy-2,7-Dimethyl-4-Octanone**	8.46	-	-	-	0.18±0.02	-	-
**6-Methyl-5-Hepten-2-One**	9.24	-	0.22±0.02[a]	0.21±0.03[a]	-	0.13±0.02[b]	-
**1-Cyclohexyl-3-Ethoxy-Butan-2-One**	9.75	-	0.23±0.02	-	-	-	-
**7,9-di-tert-butyl-1-oxaspiro[4.5]deca-6,9-diene-2,8-dione**	34.16	0.64±0.03[a]	0.28±0.03[b]	-	-	0.21±0.02[c]	0.22±0.02[c]
	**Total**	**0.87**	**1.44**	**0.50**	**0.47**	**0.63**	**1.09**
**Others**							
**Cytidine, N-acetyl-**	3.08	-	-	-	-	0.12±0.01	-
**Methyl-d3 1-Dideuterio-2-propenyl Ether**	4.11	0.45±0.04[a]	0.25±0.02[d]	0.33±0.02[c]	-	0.39±0.02[b]	0.48±0.03[a]
**Vinyl 2-(ethoxy)ethyl**	4.35	-	0.20±0.02	-	-	-	-
**Methoxy, Phenyl-,Oxime**	7.63	-	-	-	0.11±0.01	-	-
**1 Alpha.-Terpinyl Acetate**	8.64	-	-	-	-	-	0.26±0.02
**1,2-Benzenedicarboxylic acid, bis(2-methylpropyl) ester**	9.73	-	-	-	-	-	0.23±0.02
**Oxirane, tetradecyl-**	15.02	-	0.25±0.02	-	-	-	-
**1-P-Menthen-8-YL Acetate**	19.47	-	-	0.27±0.02[a]	-	0.22±0.01[b]	-
	**Total**	**0.45**	**0.70**	**0.60**	**0.11**	**0.73**	**0.97**
**Total area percentage (%)**		**100**	**100**	**100**	**100**	**100**	**100**

*The amount of each compound group for each bread relative to peak area (%). -: *not detected*.
